# Determining Chemically and Spatially Resolved Atomic Profile of Low Contrast Interface Structure with High Resolution

**DOI:** 10.1038/srep08618

**Published:** 2015-03-02

**Authors:** Maheswar Nayak, P. C. Pradhan, G. S. Lodha

**Affiliations:** 1 Indus Synchrotrons Utilization Division, Raja Ramanna Centre for Advanced Technology, Indore-452013 (M P), India

## Abstract

We present precise measurements of atomic distributions of low electron density contrast at a buried interface using soft x-ray resonant scattering. This approach allows one to construct chemically and spatially highly resolved atomic distribution profile upto several tens of nanometer in a non-destructive and quantitative manner. We demonstrate that the method is sensitive enough to resolve compositional differences of few atomic percent in nano-scaled layered structures of elements with poor electron density differences (0.05%). The present study near the edge of potential impurities in soft x-ray range for low-Z system will stimulate the activity in that field.

Thin films and multilayers (MLs), nano-structured in one dimension, have unique optical, structural, electronic and magnetic properties with a wide range of applications^1^,^2^. Properties of these structures are strongly influenced by presence of small quantity of impurity, layer composition, interfacial microstructure and chemical nature^3^,^4^,^5^. Low electron density contrast (EDC) structures are of enormous interest. For *e.g.*, in Co/Cu magnetic MLs, presence of small magnetic impurity (*e.g.,* Ni) concentrations in the nonmagnetic (Cu) layer brings drastic changes in magnetic coupling and magnetoresistance (ref. 4). Similarly, as the size of the semiconductor structure decreases, the dopant distribution−which plays a fundamental role in determining the properties−become narrower (~nm). Establishing a microscopic picture for fundamental understanding of this narrow low-atomic number (Z) doping layer require spatial and chemical characteristics on the atomic scale^6^,^7^,^8^. Recently, growth of graphene on SiC and SiO_2_ is of interest due to its unique physical and electronic properties and find potential applications^9^. Because of its importance, numerous efforts have been invested to characterize low contrast underlying interface structure and chemical nature which strongly influences growth and properties^10^,^11^,^12^ using different techniques^13^,^14^. Again, low EDC structures with low-Z/low-Z combinations^15^,^16^ and in particular Si/B_4_C structure is of current interest due to potential application in emerging fields like astrophysics^17^, low bandpass filter^18^ and electronic applications^19^, where the properties of these structures are strongly affected by the interfacial microstructure and atomic distribution. However, accurate understanding of atomic distribution and microstructure remain uncertain due to low contrast problem (ref. 18). Progress in understanding and predicting the properties relies on quantitative information about the distribution of these parameters at the atomic scale. So, it is clear that chemically resolved small atomic concentration and their spatial distribution across nano-scaled buried interfaces of low EDC are interesting and important aspects, and those need to be investigated.

Despite important scientific interest, quantitative precise measurement of such informations at deeply embedded interfaces are very scarce owing to the fact that there are not many techniques available to measure such a small quantity of chemically resolved atomic composition profile and microstructure wherever layer thicknesses of the order of ~ nm are involved. A combination of conventional hard x-ray reflectivity (XRR) and x-ray standing waves (XSW) analysis has been employed to quantify such a distribution^20^,^21^ with a precision of ~2 atomic percent (at.%) and depth resolution of ~0.1 nm^22^ generally from high contrast periodic ML. However, difficulty arises in XRR to probe microstructure when the EDC at an interface is low (Δ*ρ*/*ρ* ≤ 5%)^23^, and to extract the layer composition. For *e.g.*, in a Pt/C ML, even a 15% change in the electron density of the C-layers (say due to Pt diffusion into the C-layers) does not produce a significant change in XRR (ref. 20). So, combined XSW-XRR techniques are restricted in their success owing to lack of sensitivity in structures like: (i) For non-periodic structure and/or with low-Z materials where x-ray fluorescence signal is very weak. (ii) For low contrast interfaces because of contrast limit of XRR. Recently, a nice study has been done on mono-layers of graphene/SiC (0001) interface^24^ using XSW-excited photoelectron spectroscopy, however it is a surface sensitive (~1–10 nm) technique. Indeed, here we present using resonant soft x-ray reflectivity (R-SoXR), a method that can overcome previous limitations owing it's excellent chemical sensitivity to low-Z materials, high contrast variation and high resolution. R-SoXR has been used for studying polymeric & organic materials^25^,^26^,^27^,^28^,^29^,^30^, ionic liquid^31^, electronic and structural analysis of hard matter^32^,^33^,^34^ and magnetization in magnetic structures^35^,^36^,^37^. However, very little is known about it's utility to precisely measure chemically and spatially resolved atomic distribution profile across low contrast and low-Z interface structure.

In this letter, precise quantitative measurements of both chemically selective atomic concentration and their spatial distributions along the microstructure of underlying Si/B_4_C interfaces are presented. We observe that there is a chemical change in B_4_C and we are in position to resolve differences of few at.% of compositional variation and their spatial distribution which ultimately enables for construction of highly chemically resolved interfacial map.

## Results

### Hard x-ray reflectivity

**Figure 1:**
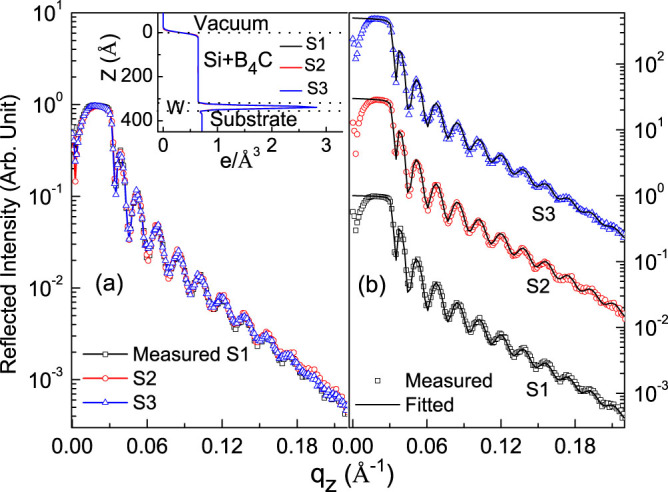
(a) Overlap of measured hard XRR of three samples (S1, S2 and S3) upto q_z_ = 0.23. (b) Measured along with fitted XRR profile (vertically shifted). Inset shows EDP obtained from best-fit hard XRR results.

Thin film samples are fabricated with varying position of B_4_C layer (30Å) in Si thin film of thickness 300 Å. B_4_C is at top, middle and bottom of Si layer for sample 1 (S1), sample 2 (S2) and sample 3 (S3), respectively. In all samples, a W layer of thickness 10 Å is deposited just above the Si substrate to provide an optical contrast between substrate and the film. Prior to R-SoXR measurements, hard XRR measurements are done using Cu *K_α_* source and data are plotted upto q_z_ = 0.23 (figures 1(a) and (b)) to compare with q_z_-range of R-SoXR measurements. Measured profiles of three samples with varying position of B_4_C layer in Si clearly appear very similar (figure 1(a)). Inset of figure 1(a) shows also nearly identical electron density profiles (EDP) obtained from best-fit results of XRR of S1, S2 and S3 (figure 1(b)). The fitted profile matches well with the measured curve by considering Si and B_4_C as a single layer having total thickness of 333 ± 1 Å; and mass density ~90 ± 2% of bulk value of Si with rms roughness ~8 ± 0.5, 7.5 ± 0.5 and 9 ± 0.5 Å; for samples S1, S2 and S3, respectively. W layer thickness is 10.5 ± 0.5 Å with mass density ~92 ± 2% of bulk value having rms roughness ~6 ± 0.5 Å for all samples. The rms roughness of the substrates is ~4.5 ± 0.5 Å. Thus, conventional XRR is not sensitive to Si/B_4_C interface having low EDC, Δ*ρ*/*ρ* = 0.05%, and to compositional changes in the film, due to low contrast and lack of element-specificity.

### Sensitivity of resonant reflectivity to low contrast interface

**Figure 2:**
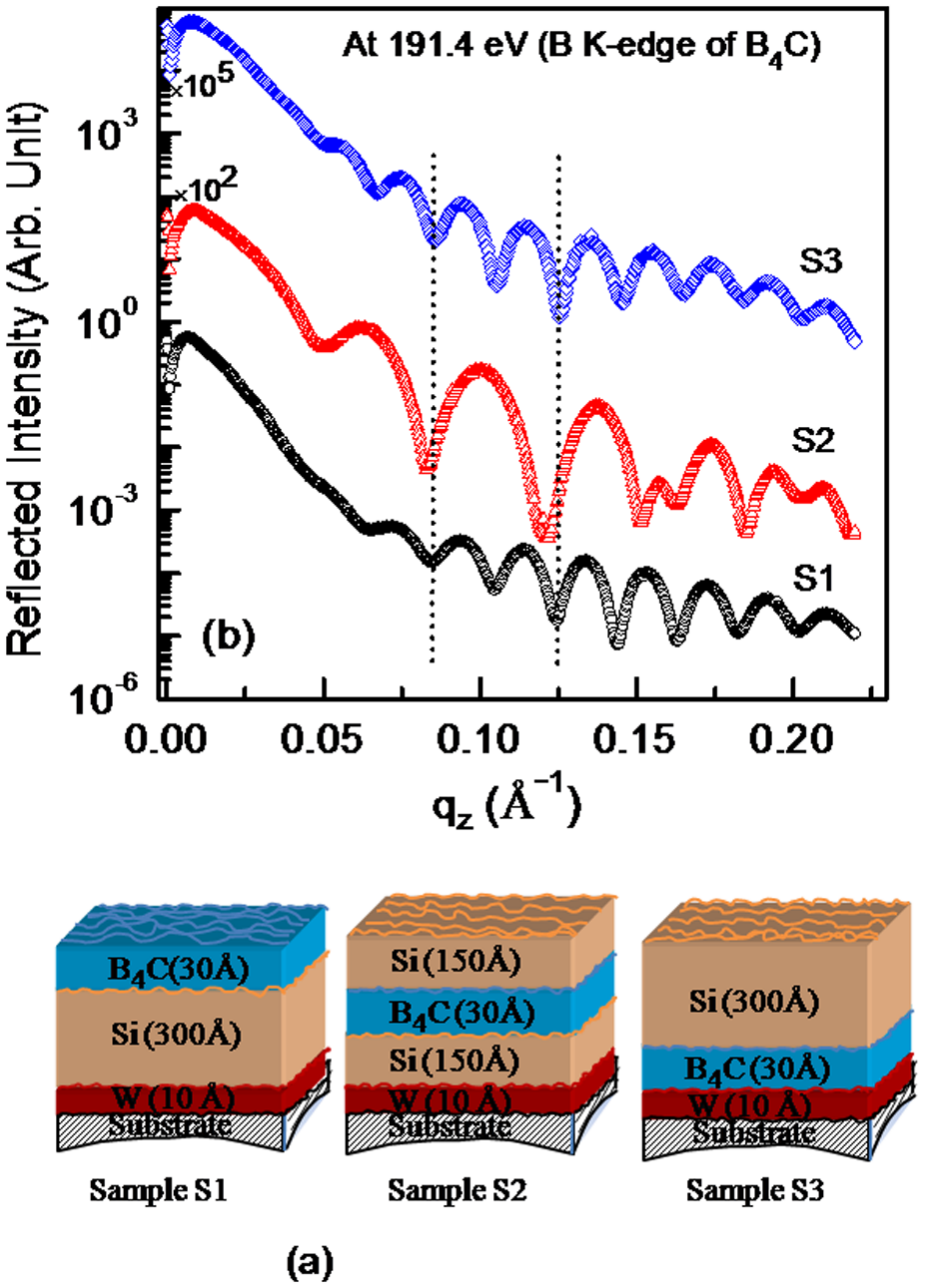
(a) Schematic diagram of three fabricated samples with varying spatial positions of B_4_C layer. (b) Measured R-SoXR profiles at a selected energy of 191.4 eV (B K-edge of B_4_C).

**Figure 3:**
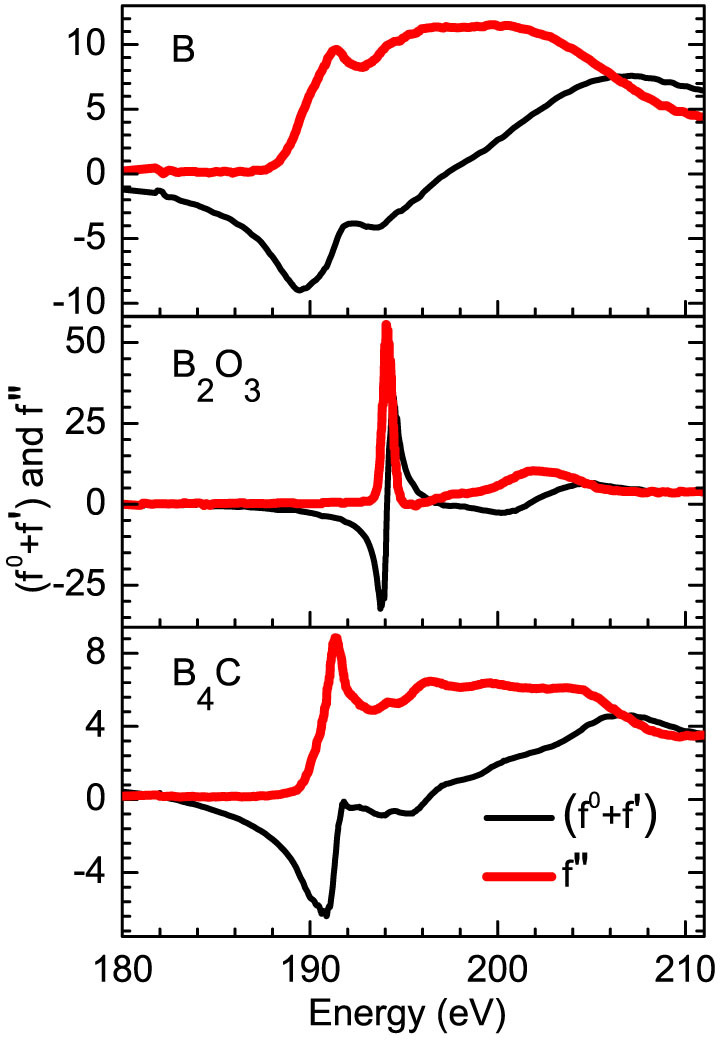
Measured ASF of B, B_4_C and B_2_O_3_ near boron K-edge to understand and correlate with the observed R-SoXR profiles.

Sensitivity of resonant reflectivity to low contrast Si/B_4_C interface is demonstrated by performing repeated measurements at a selected energy of 191.4 eV (B K-edge of B_4_C) (figure 2 (b)). Figure 2(a) illustrates schematic diagram of three deposited samples S1, S2 and S3 with different spatial positions of B_4_C layer. To understand the observed scattered profiles for chemically selective atomic distribution analysis, the measured atomic scattering factor (ASF) of B, B_4_C and B_2_O_3_ near boron K-edge are shown in figure 3. At this specified energy of 191.4 eV, ASF of B_4_C has a strong variation (figure 3). The strong modulations in reflected spectra (figure 2 (b)) is due to major reflection contribution from Si/B_4_C interface apart from contributions from other interfaces. Thus the measured profiles of three samples are significantly different, as the spatial position of B_4_C layer changes in Si film. The origin of the long period oscillations in the reflectivity curve for S2 is related to the strong optical contrast at Si/B_4_C where B_4_C is sandwich between two Si layers resulting smaller individual Si thickness. Two vertical dotted lines mark how the period of oscillations gets modulated as position of the B_4_C layer varies in Si film. This provides an experimental evidence for sensitive of R-SoXR to the spatial variation of a low contrast interface. The results demonstrated here with Δ*ρ*/*ρ* = 0.05% as an example, has two orders of magnitude better EDC sensitivity compared to conventional hard XRR.

### Spectroscopic like information using resonant reflectivity

**Figure 4:**
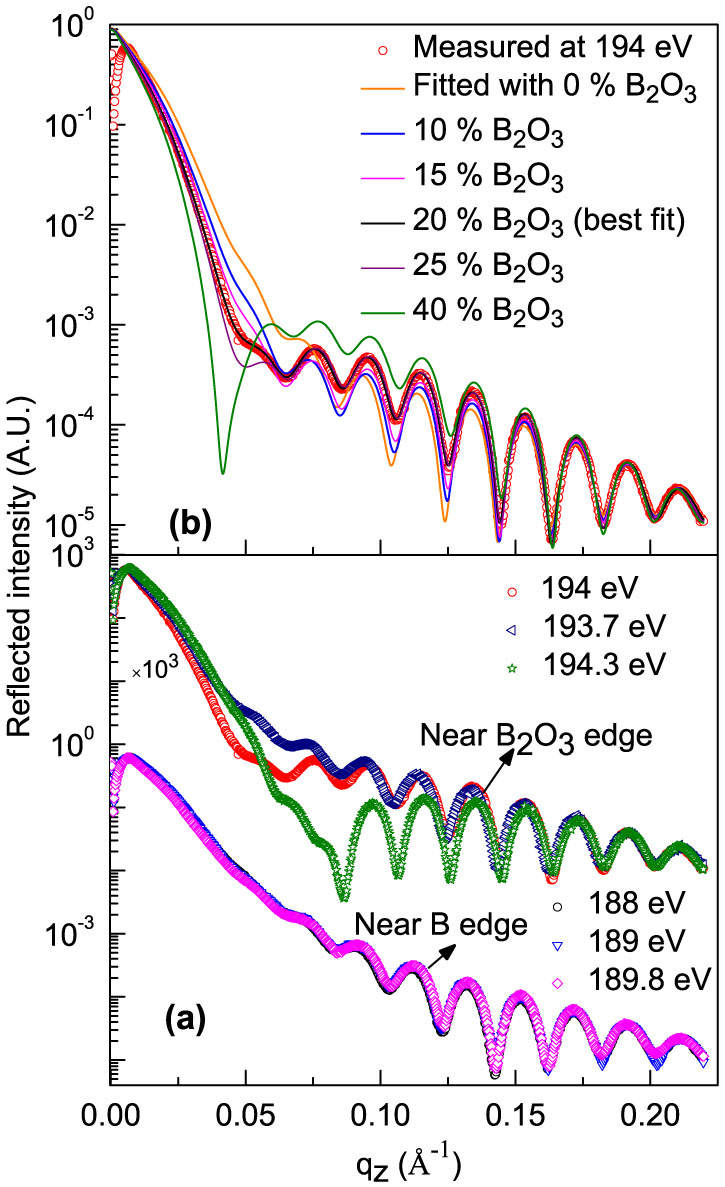
(a) Measured R-SoXR profiles of sample S1 at selected energies near B K-edge of both B and B_2_O_3_. (b) Measured R-SoXR profile of S1 at a selected energy of 194 eV (near B_2_O_3_ edge) along with fitted profiles with varying atomic percent of B_2_O_3_ in the top B_4_C layer.

In ion beam sputter deposited B_4_C films, there may be a partial decomposition of B_4_C to elementary boron (B). The elementary B on the surface is likely to react with oxygen when it is exposed to ambient conditions. The question arises whether there is a chemical change in the B_4_C layers, and if so, to quantity it and to determine the elemental distribution from the surface down to a depth of ~300 Å. To obtain spectroscopic like information of whether chemical changes exist in the B_4_C layer or not, R-SoXR measurements are performed at selected energies near the respective absorption edge of boron and it's all the possible compounds. Figure 4(a) demonstrates experimental evidence of the presence of chemical changes in sample S1. The measurements are performed at B K-edge of both elementary B (~189.5 eV) and B_2_O_3_ (~194.1 eV). Near B edge, three energies of 188, 189 and 189.8 are chosen. At these energies the ASF undergoes strong variation for boron but not for B_2_O_3_ (figure 3). If the film contains elementary B within penetration depth of x-ray (for e.g., at 189.8 eV, penetration depth in B is ~500 Å), it will produce a strong modulation in reflected spectra as incident energy is varied in these range. However, the measured reflected spectra are clearly appear very similar near B K-edge of elementary B (figure 4(a)). This observation corroborates no elementary B is present in sample S1. The upper limit of elementary boron in sample S1 is ~3% consistent with the measurement. Similarly, to confirm the presence of B_2_O_3_ in sample S1, R-SoXR measurements are performed at three selected energies of 193.7, 194 and 194. 3 eV across the strong B K-absorption edge of B_2_O_3_. At these energies the ASF of B_2_O_3_ undergoes strong variation but elementary boron exhibits nearly a flat optical response (figure 3). The scattering strength of B_2_O_3_, 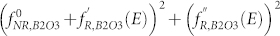, at energies 193.7, 194 and 194. 3 eV are ~1400, 2940 and 2079, respectively which are more than three orders of magnitude higher than that of away from absorption edge (for e.g., 0.4 at 185 eV). Thus, near the edge, B_2_O_3_ provides enhanced and tunable scattering. In figure 4(a), near B K-edge of B_2_O_3_, as the energy changes from 193.7 to 194.3 eV, the measured R-SoXR curves undergo strong variation with significant change in the amplitude as well as shape of the oscillations. This corroborates presence of B_2_O_3_ in sample S1. These experimental results provide evidence of the chemical changes in B_4_C layer which may be due to decomposition of some of B_4_C during deposition. In sample S1, all the decomposed B atoms in the top B_4_C layer are fully oxidized.

### Chemically selective quantitative atomic profile

To quantify atomic percent of B_2_O_3_ and it's spatial distribution in B_4_C layer of sample S1, R-SoXR measured data along with fitted profiles with different models are shown in figure 4(b). The measured data are fitted by slicing B_4_C layer with different thicknesses and atomic compositions to account spatial variation of at. % of B_2_O_3_ within B_4_C layer. However, the best-fit data matches well with the experimental data with uniform distribution model. The layer thickness and roughness obtained by simultaneous fitting measured data at different selected energies near B K-edge of B_2_O_3_ are kept constant. The optimized value for thickness (roughness) of Si and B_4_C layers are 302 Å (9 Å) and 31 Å (8 Å), respectively. Figure 4(b) shows the variation of fitted profiles with the measured R-SoXR curve (at energy 194 eV) when the content of at. % of B_2_O_3_ in B_4_C layer is varied. As at. % of B_2_O_3_ is varied from 0 to 40%, the reflected profile undergoes strong modulation producing changes in both the amplitude and shape of the oscillations envelope. Here, it is mentioned that while structural parameters are linked to the periods of the oscillations in the reflected profile, parameters of the atomic composition of the resonating atom/compound are closely related to the amplitudes and shape of the oscillations envelope. Even by mixing 5% of B_2_O_3_, brings significant change in optical properties of the B_4_C layer (*e.g*., *δ* changes from −4.53 × 10^−4^ to −8.33 × 10^−4^ and β changes from 2.62 × 10^−3^ to 3.41 × 10^−3^) at 194 eV, which brings significant changes in reflected spectra. The scattering contrast at interface, (Δ*δ*)^2^ + (Δ*β*)^2^, which is proportional to scattering intensity undergoes significant and tunable enhancement. In figure 4 (b), the fitted profile with 20 at. % of B_2_O_3_ in top B_4_C layer is well matches the measured curve. The sharp and strong resonance effect provides more accuracy. The result clearly reveals resonant reflectivity is a highly sensitive technique to quantify atomic composition within a few at. % of precision.

**Figure 5:**
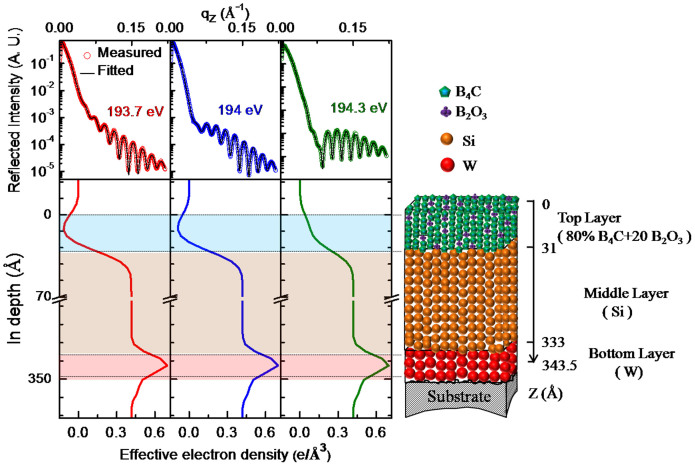
Top panel shows measured R-SoXR profiles along with best-fit data of Sample S1 at selected energies near B K-edge of B_2_O_3_. The corresponding bottom panel shows effective EDP. The schematic diagram at right side shows an illustrative the vertical depth profile of composition profile modeled for real structure in sample S1. Size of balls is not scales to actual size of atoms and compounds.

The effective EDP (bottom panel of figure 5) is obtained from the best-fit R-SoXR curve (top panel of figure 5) at three different selected energies. The EDP undergoes gradual variation at the interfaces and is sensitive to Si/B_4_C interface. The EDP profiles clearly show that the position of B_4_C layer is at top of Si in sample S1. The EDP of B_4_C layer containing B_2_O_3_ undergoes significant change as the energy is tuned near B K-edge of B_2_O_3_. A schematic diagram representing model of vertical atomic composition distribution in different layers obtained from best-fit R-SoXR results is shown in right hand side of figure 5. The best-fit results of sample S1 are: thickness (roughness) of W, Si and B_4_C layers as 10.5 ± 0.5 Å (6 ± 0.5 Å), 302 ± 1 Å (9±0.5Å) and 31 ± 0.5 Å (8 ± 0.5 Å), respectively. The best-fit results also reveal that the top B_4_C layer is composed of ~80 ± 3% of B_4_C and ~20 ± 3% of B_2_O_3_.

**Figure 6:**
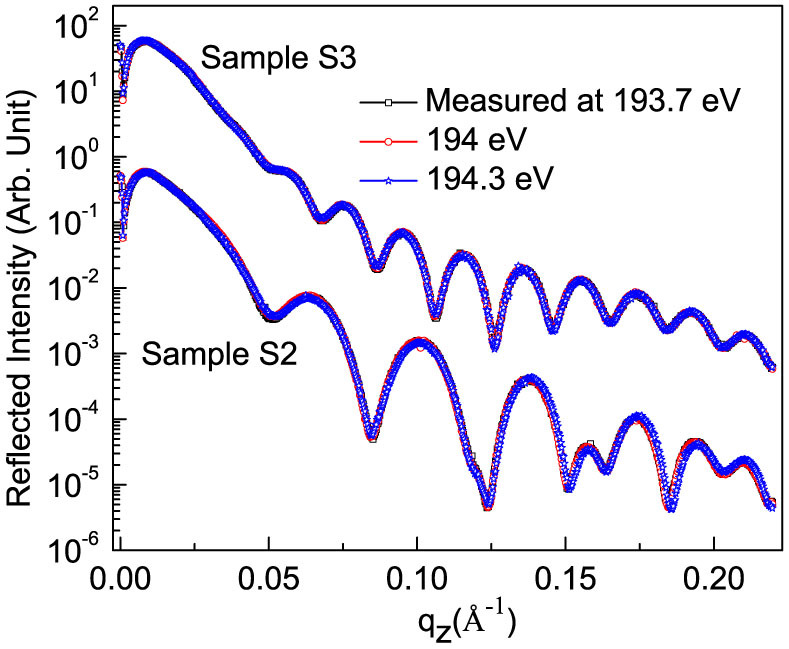
Measured R-SoXR profiles at selected photon energies near B K-edge of B_2_O_3_ of samples S2 and S3.

**Figure 7:**
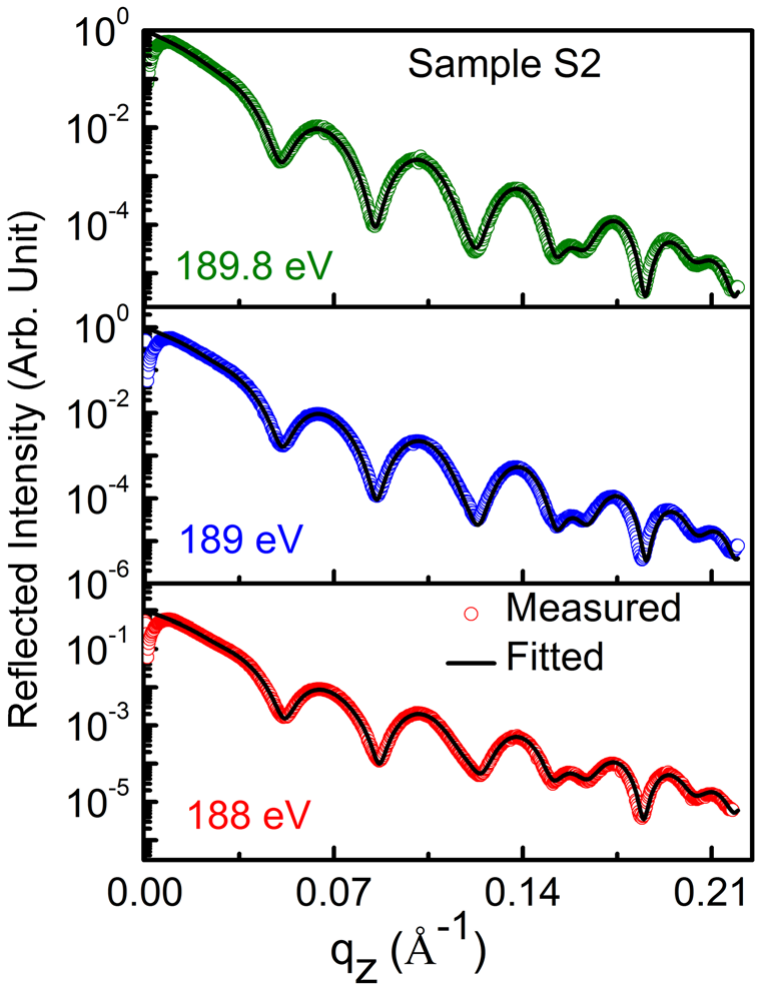
Measured R-SoXR curves along with best-fit profiles of Sample S2 at selected energies near B K-edge of elementary B.

Similar to quantitative determination of atomic profile along with microstructure for sample S1, those of samples S2 and S3 have been also determined. The procedure for data analysis for samples S2 and S3 is similar to that of S1. In order to find spectroscopic like information of whether B_2_O_3_ is present in the samples S2 and S3 or not, R-SoXR measurements are performed across the very strong and sharp B K-absorption edge of B_2_O_3_ (figure 6). However, the measured R-SoXR profiles are nearly identical in nature at three selected energies of 193.7, 194 and 194.3 eV for both S2 and S3. R-SoXR measured data are consistent with repeating the measurements three times. This confirms B_2_O_3_ is not present in samples S2 and S3. The upper limit of B_2_O_3_ in sample S2 and S3 is ~3% consistent with measurement. The presence of elementary boron in sample S2 is confirmed by significant variation of measured R-SoXR profiles near the B K-edge of elementary boron at three selected energies of 188, 189 and 189.8 eV (figure 7). In figure 7, the fitted profile well matches the measured curve for three different energies. The best-fit results of sample S2 are: thickness (roughness) of W, bottom Si, B_4_C and top Si layers as 10.5 ± 0.5 Å (6 ± 0.5 Å), 151 ± 1 Å (7.5 ± 0.5 Å), 31 ± 0.5 Å (5 ± 0.5 Å) and 151 ± 1 Å (7.5 ± 0.5 Å), respectively. The best-fit results reveal that B_4_C layer is composed of ~80 ± 3% of B_4_C and ~20 ± 3% of B. Similarly for sample S3, the best-fit results of R-SoXR measurements near B K-edge of elementary B are obtained as: thickness (roughness) of W, B_4_C and top Si layers are 10.5 ± 0.5 Å (6 ± 0.5 Å), 31 ± 0.5 Å (5 ± 0.5 Å) and 302 ± 1 Å (9 ± 0.5 Å), respectively. The best-fit results reveal that B_4_C layer is composed of ~80 ± 3% of B_4_C and ~20 ± 3% of B. This clearly demonstrates the sensitivity of our measurements and novelty in the approach.

## Conclusion

In conclusion, we precisely measured chemically and spatially resolved atomic distribution profile with high resolution along with microstructure of the low contrast buried interfaces critical for nano-scaled layered structure devices. In prospective, methodology introduced here can be readily generalized to other complex multi-component interfaces. Structures up to several tens of nanometer thickness relevant to many scientific and technological problems can be studied. Such quantitative precise measurements help to understand properties of layered structures associated with chemically resolved interface map.

## Methods

All the thin film samples are fabricated using ion beam sputtering system with base pressure of ~2 × 10^−8^ mbar. R-SoXR measurements are carried out in the s-polarized geometry using reflectometry beamline on Indus-1 synchrotron^38^. R-SoXR data are fitted using Parratt formalism^39^. R-SoXR data analysis requires a precise value of near edge optical constants, δ and β (refractive index n = 1 − δ + iβ), of materials and hence atomic scattering factor, 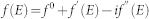. Limitation of Henke optical data^40^ is the lack of fine spectral details to describe optical properties near absorption edges and hence requires precise measured optical constants for R-SoXR analysis. Precise near edge optical constants for B_4_C, B and B_2_O_3_ are obtained using the measured total electron yield absorption data^41^ and using Kramers-Kronig relation. For the model fitting of R-SoXR data, the structural parameters such as substrate roughness, and W layer thickness, density and roughness obtained from hard XRR are used. Starting guess for B_4_C and Si layer thickness are used as per deposited value. Measured optical constants are used for resonating materials and that of non-resonating materials are taken from Henke *et al*. (ref. 40). The mass densities used for calculation of optical constants of B, B_4_C, B_2_O_3_, Si, and W are 2.34, 2.52, 2.46, 2.33 and 19.3 gm/cm^3^, respectively.

## Author Contributions

M.N. took part in conceiving the idea and performed experiments; M.N., G.S.L. and P.C.P. discussed the results; M.N. wrote the manuscript; All authors reviewed the manuscript.
