# Corrigendum: Determining Chemically and Spatially Resolved Atomic Profile of Low Contrast Interface Structure with High Resolution

**DOI:** 10.1038/srep27322

**Published:** 2016-07-11

**Authors:** Maheswar Nayak, P. C. Pradhan, G. S. Lodha, A. Sokolov, F. Schäfers

## Introduction

Scientific Reports 5: Article number: 861810.1038/srep08618; published online: 03022015; updated: 07112016

It was brought to the authors’ attention that the original paper contains the following errors. (i) We reported a 0.05% electron density contrast between silicon and boron carbide. There was a calculation error in computing this number and the correct contrast is 0.5%. This is one order of magnitude lower than what can be studied using hard x-ray reflectivity. Therefore, with this revised electron density contrast value, the proposed methodology is still valid. (ii) Numerical errors were made during the conversion of the measured angular reflectivity to q_z_ (4π sinθ/λ). To revalidate the proposed methodology, we have performed fresh measurements on similar new samples. The fresh soft x-ray resonant reflectivity measurements were done using the Optics Beamline at the BESSY storage ring which has a better energy resolution (E/ΔE ≅ 670), smaller vertical angular divergence (0.5 mrad), larger photon flux (~1.4 × 10^10^) and accessible q-space compared to the measurements reported in the original paper using the Indus -1 reflectivity beamline. The results are presented below and the methodology and the conclusion reported in the original paper still stand.

### Hard x-ray reflectivity

Thin film samples are fabricated with varying position of B_4_C layer (40 Å) in Si thin film of thickness 300 Å. The samples are fabricated using electron beam evaporation. Elementary boron is incorporated into B_4_C layer by co-deposition. B_4_C is at top, middle and bottom of Si layer for sample 1 (S1), sample 2 (S2) and sample 3 (S3), respectively. In all samples, a W layer of thickness 10 Å is deposited just above the Si substrate to provide an optical contrast between substrate and the film. Prior to R-SoXR measurements, hard XRR measurements are done using Cu *K*_*α*_ source. Hard XRR profile of all three samples are measured and fitted up to q_z_ = 0.42 Å^−1^ (theta = 3 degree). However, hard XRR profile are plotted up to q_z_ = 0.22 Å^−1^ (theta = 1.545 degree) [Figure 1(a,b)]. Measured profiles of three samples with varying position of B_4_C layer in Si clearly appear very similar [Figure 1(a)]. Inset of Figure 1(a) shows nearly identical electron density profiles (EDP) obtained from best-fit results of XRR of S1, S2 and S3 [Figure 1(b)]. The fitted profile matches the measured curve by considering Si and B_4_C as a single layer. The total thickness of (Si + B_4_C) is 350 ± 1, 352 ± 1 and 353 ± 1 Å; and mass density is about 95 ± 2% of bulk value of Si with rms roughness = 7.5 ± 0.5, 6.5 ± 0.5 and 7 ± 0.5 Å; for samples S1, S2 and S3, respectively. W layer thickness is ~10 Å having rms roughness = 3.5 ± 0.5, 4 ± 0.5 and 4.5 ± 0.5 Å; for samples S1, S2 and S3, respectively. The rms roughness of the substrates is 4.5 ± 0.5 Å. A silicon oxide layer of thickness ~15.5 Å is considered above the silicon substrate. Thus, conventional XRR is not sensitive to Si/B_4_C interface having low electron density contrast (EDC), ∆ρ/ρ = 0.5%, and to compositional variation in the film, due to low contrast and lack of element-specificity.

### Sensitivity of resonant reflectivity to low contrast interface

Sensitivity of resonant reflectivity to low contrast Si/B_4_C interface is demonstrated by performing measurements at a selected energy of 191.4 eV (B K-edge of B_4_C) [Figure 2(b)]. Soft x-ray reflectivity measurements are carried out in the s-polarization geometry using the Optics Beamline at the BESSY-II storage ring^1^,^2^. The measurements were done with a better energy resolution, photon flux, accessible q-space and lower angular divergence than the measurements presented in the original paper. For the soft x-ray measurements, the data are collected up to theta = 89.2 degree. The reflectometer used was specially designed for measurements in near-normal incidence geometry. A GaAsP-photodiode of 4 × 4 mm2 acceptance area, surrounded by a support of 2 mm diameter at a distance of 310 mm from the sample was used. The minimum angle to normal is thus atan(4/310) = 0.74°, corresponding to 89.26° grazing angle. Figure 2(a) illustrates schematic of three deposited samples S1, S2 and S3 with different spatial positions of B_4_C layer. To understand the observed scattered profiles for chemically selective atomic distribution analysis, the measured atomic scattering factor (ASF) of B, B_4_C and B_2_O_3_ near boron K-edge are shown in Figure 3. At this specified energy of 191.4 eV, ASF of B_4_C has a strong variation [Figure 3]. The strong modulations in reflected spectra [Figure 2(b)] is due to major reflection contribution from Si/B_4_C interface apart from contributions from other interfaces. Due to the contribution of the reflection from the Si/B_4_C interface, the three different layer structures of three samples (S1, S2 and S3) exhibit significantly different measured profiles with a strong modulation, as the spatial position of B_4_C layer changes in Si film. Two vertical dotted lines mark how the period of oscillations gets modulated as position of the B_4_C layer varies in Si film. This provides an experimental evidence for sensitivity of resonant soft x-ray reflectivity (R-SoXR) to the spatial variation of a low contrast interface. The results demonstrated here with ∆ρ/ρ = 0.5% as an example, has one order of magnitude better EDC sensitivity compared to conventional hard XRR^3^.

### Spectroscopic information using resonant reflectivity

To determine the spectroscopic information using R-SoXR, elementary boron is introduced in B_4_C layer by co-deposition using electron beam evaporation method. R-SoXR measurements are performed at selected energies near the respective absorption edges of boron and the compounds of boron. Figure 4(a) demonstrates experimental evidence of the presence of chemical changes in sample S1. The measurements are performed at B K-edge of both elementary B (~189.5 eV) and B_2_O_3_ (~194.1 eV). Near B K-edge of B_2_O_3_, four energies of 193.7, 194, 194. 3 and 194.6 eV are chosen across the edge. At these energies the ASF undergoes strong variation for B_2_O_3_ but elementary boron exhibits nearly a flat optical response [Figure 3]. If the film contains B_2_O_3_ within penetration depth of x-ray, it will produce a strong modulation in reflected spectra as incident energy is varied in these ranges. The measured reflection spectra clearly appear very similar near B K-edge of B_2_O_3_ [Figure 4(a)]. This observation corroborates that no B_2_O_3_ is present in sample S1. Similarly, to confirm the presence of B in sample S1, R-SoXR measurements are performed across the B K-absorption edge of B at selected energies of 185, 186,187, 188, 189, 190.7 and 191.4 eV [Figure 4(a)]. At these energies the ASF undergoes strong variation for boron but not for B_2_O_3_ [Figure 3]. Near the edge, B provides enhanced and tunable scattering. B_4_C also exhibits variation of ASF with energy towards higher side with respect to elementary B. However, the magnitude of variation of ASF is more in B than B_4_C due to stronger resonance enhancement of elementary B than B in B_4_C. The observed changes in the reflected profile at the selected energies across the B K-edge of elementary boron can be due to contribution of both kinds of atoms. At the lower energy side, the variation in the measured profiles is dominated by the contribution of elementary boron. The contribution of B_4_C starts at higher energy along with elementary boron. This corroborates the presence of B in sample S1. In the original paper, the elementary boron was not detected in S1, as elementary boron is fully oxidized when exposed to ambient condition. Whereas in the fresh sample S1 (in corrigendum), the elementary boron in the top B_4_C layer is not oxidized because of a contaminated carbon layer at the top. This contaminated carbon layer most likely prevents elementary boron in the top B_4_C layer to be oxidized in fresh sample S1.

### Chemically selective quantitative atomic profile

To quantify the atomic percentage of B and the spatial distribution in B_4_C layer of sample S1, R-SoXR measured data along with fitted profiles with different models are shown in Figure 4(b). The measured data are fitted by slicing B_4_C layer with different thicknesses and atomic compositions to account for a spatial variation of at. % of B within B_4_C layer. However, the best-fit data matches well with the experimental data with uniform distribution model. The layer thickness and roughness obtained by simultaneous fitting measured data at different selected energies near B K-edge of B are kept constant. The optimized value for thickness (roughness) of Si and B_4_C layers are 294 Å (5 Å) and 42 Å (13 Å), respectively. An intermixing layer at the Si/B_4_C interface is considered with thickness 11.5 Å and roughness 7.5 Å. A carbon contaminated layer with thickness 11.5 Å and roughness 6.5 Å is also considered at the top of B_4_C layer. Figure 4(b) shows the variation of fitted profiles with the measured R-SoXR curve (at energy 190.7 eV) when the content of atomic % of B in B_4_C layer is varied. As B is varied from 0 to 40%, the reflected profile undergoes strong modulation producing changes in both the amplitude and shape of the oscillations envelope. Here, it is mentioned that while structural parameters are linked to the periods of the oscillations in the reflected profile, parameters of the atomic composition of the resonating atom/compound are closely related to the amplitudes and shape of the oscillations envelope. Resonant x-ray reflectivity has excellent chemical sensitivity to the resonating atom along with their spatial distribution. This high sensitivity determines the chemically and spatially resolved atomic profile within the nanometer range with a very tiny volume of contributing material. The significant change in reflectivity profile at around q = 0.05 Å^−1^ by varying percent of elementary B in Figure 4(b) could be due to type of layer structure chosen in the thin film for the case study, the optical properties of the resonating atom and change in optical contrast by varying with atomic percent of B. The changes in the values of atomic scattering factor/optical constant (δ and β) by incorporation of different percent of B in B_4_C layer are as follows: At 190.7 eV, the values of δ and β of B_4_C layer with 0%, 10%, 15%, 20%, 25% and 40% of B are as follows: −3.17 × 10^−3^ and 2.29 × 10^−3^, −4.72 × 10^−3^ and 4.09 × 10^−3^, −5.49 × 10^−3^ and 4.99 × 10^−3^, −6.27 × 10^−3^ and 5.89 × 10^−3^, −7.04 × 10^−3^ and 6.78 × 10^−3^, and −9.36 × 10^−3^ and 9.48 × 10^−3^, respectively. Even by mixing 5% of B, brings significant changes in the optical properties of the B_4_C layer, which brings significant changes in the reflected spectra as well. The scattering contrast at interface, 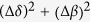, which is proportional to scattering intensity undergoes significant and tunable enhancement. In Figure 4(b), the fitted profile with 20 atomic % of B in the B_4_C layer matches the measured curve well. The result clearly reveals resonant reflectivity is a highly sensitive technique to quantify atomic composition within a few atomic % of the precision.

The effective EDP [bottom panel of Figure 5] is obtained from the best-fit R-SoXR curve [top panel of Figure 5] at three different selected energies. The EDP undergoes gradual variation at the interfaces and is sensitive to the Si/B_4_C interface. The EDP profiles clearly show that the position of B_4_C layer is at top of Si in sample S1. The EDP of B_4_C layer containing B undergoes significant changes as the energy is tuned near the B K-edge of elementary boron due to the contribution of both types of B atoms (i.e. elementary B and B in B_4_C) at these energies. A schematic model of the vertical atomic composition distribution in different layers obtained from best-fit R-SoXR results is shown in the right hand side of Figure 5. The best-fit results of sample S1 are: average thickness (roughness) of W, Si, interlayer (mixed layer) (B_4_C-on-Si), B_4_C and the top contaminated carbon layers as 8 ± 1 Å (3.5 ± 0.5 Å), 294 ± 1 Å (5 ± 0.5 Å), 11.5 ± 1 Å (7.5 ± 0.5 Å), 42 ± 1 Å (13 ± 0.5 Å) and 11.5 ± 1 Å (6.5 ± 0.5 Å), respectively. The best-fit results also reveal that the B_4_C layer is composed of 80 ± 3% of B_4_C and 20 ± 3% of B. The interlayer (mixed layer) is composed of 80% of Si and 20% of (80% B_4_C + 20% B).

Similar to quantitative determination of the atomic profile along with microstructure for sample S1, those of samples S2 and S3 have been also determined. The procedure for data analysis for samples S2 and S3 is similar to that of S1. In order to find spectroscopic information of whether B_2_O_3_ is present in the samples S2 and S3 or not, R-SoXR measurements are performed across the very strong and sharp B K-absorption edge of B_2_O_3_ [Figure 6]. However, the measured R-SoXR profiles are nearly identical in nature at four selected energies of 193.7, 194, 194.3 and 194.6 eV for both S2 and S3. This confirms that B_2_O_3_ is not present in samples S2 and S3. The presence of elementary boron in sample S2 is confirmed using the procedure followed for sample S1 (discussed earlier) by performing R-SoXR measurements across the B K-edge of elementary boron at the selected energies of 185, 186, 187, 188, 190.7 and 191.4 eV. To quantify the atomic % of B and the spatial distribution in B_4_C layer of sample S2, R-SoXR measured data along with best-fit profiles at three selected energies of 188, 190.7 and 191.4 eV are shown in Figure 7. The best-fit results of sample S2 are: average thickness (roughness) of W, Si, interlayer layer I (B_4_C-on-Si), B_4_C, interlayer II (Si-on-B_4_C) and Si layers as 8 ± 1 Å (4 ± 0.5 Å), 138 ± 1 Å (8.5 ± 0.5 Å), 13 ± 1 Å (4 ± 0.5 Å), 41 ± 1 Å (6.5 ± 0.5 Å), 13 ± 1 Å (5.5 ± 0.5 Å) and 148 ± 1 Å (7 ± 0.5 Å), respectively. The best-fit results also reveal that the B_4_C layer is composed of 80± 3% of B_4_C and 20 ± 3% of B. The interlayer (mixed layer) is composed of 80% of Si and 20% of (80% B_4_C + 20% B).

Similarly for sample S3, the best-fit results of R-SoXR measurements near the B K-edge of elementary B are obtained as: average thickness (roughness) of W, B_4_C, interlayer (Si-on-B_4_C) and Si layers as 8 ± 1 Å (5 ± 0.5 Å), 41 ± 1 Å (5.5 ± 0.5 Å), 12 ± 1 Å (6 ± 0.5 Å) and 301 ± 1 Å (7.5 ± 0.5 Å), respectively. The best-fit results also reveal that the B_4_C layer is composed of 80± 3% of B_4_C and 20 ± 3% of B. The interlayer (mixed layer) is composed of 80% of Si and 20% of (80% B_4_C + 20% B).

### Energy resolution of the measurements

The energy resolution (E/ΔE) for the energy scan to determine (f^0^ + f′) and f″ values is about 1000 at 200 eV. The energy resolution available for the angular scan, in the original paper is about 250 at 190 eV and in the corrigendum is ~670 at 190 eV with spectral impurity ~0.1%. In the original paper, due to intensity reasons the energy resolution used for the angular scan was poorer than for the energy scan. This may lead to some uncertainty for the determination of the composition. In the original paper, to better understand the changes in reflectivity profile in the vicinity of the B K-edge of B_2_O_3_ (Figure 4(a) in the original paper) although the energy resolution was not optimum, we compare the changes in the value of (f^0^ + f′) and f″ in energy interval of 0.3 eV at selected energies, below the edge (away from the edge) and near the edge. For example, as per the energy scan, below the edge, (f^0^ + f′) and f″ of B_2_O_3_ are −1.71 and 0.488 at 188.5 eV, and −1.74 and 0.476 at 188.8 eV, respectively. Below the edge, the change in (f^0^ + f′) and f″ in energy interval of 0.3 eV is small. However, near the edge, (f^0^ + f′) and f″ of B_2_O_3_ are −30.46 and 13.89 at 193.7 eV, −16.3 and 49.25 at 194 eV, and 25.5 and 39.78 at 194.3 eV, respectively. Taking into account the broadening of the resonance for the angular scan in the original paper, near the edge, (f^0^ + f′) and f″ of B_2_O_3_ are −27.77 and 17.53 at 193.7 eV, −15.93 and 40.44 at 194 eV, and 18.25 and 38.91 at 194.3 eV, respectively. Near the edge, the change in (f^0^ + f′) and f″ in energy interval of 0.3 eV is significant. The variation of the atomic scattering factor of B_2_O_3_ near the B_2_O_3_ edge provides changes in the reflectivity profile as observed in the original paper.

A. Sokolov and F. Schäfers have been added to the author list because they contributed to the experiments reported in this Corrigendum. This has now been corrected in the HTML versions of the Article. The Author Contributions section in the HTML version now reads:

M.N. took part in conceiving the idea and performed experiments in the original paper; M.N., G.S.L. and P.C.P. discussed the results; M.N. wrote the manuscript; All authors reviewed the manuscript; In the corrigendum, A. S. And F. S. played a key role in the soft x-ray measurements and optimization of the beamlines for these measurements; All the authors discussed the results in preparing the scientific contents of the manuscript; M.N. wrote the manuscript; All authors reviewed the manuscript.

## Figures and Tables

**Figure 1:**
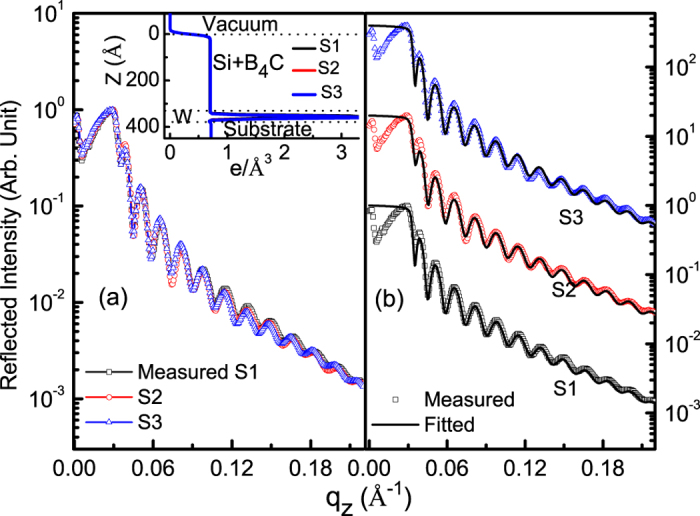
(**a**) Overlap of measured hard XRR of three samples (S1, S2 and S3) up to q_z_ = 0.22. (**b**) Measured along with fitted XRR profile (vertically shifted). Inset shows EDP obtained from best-fit hard XRR results.

**Figure 2:**
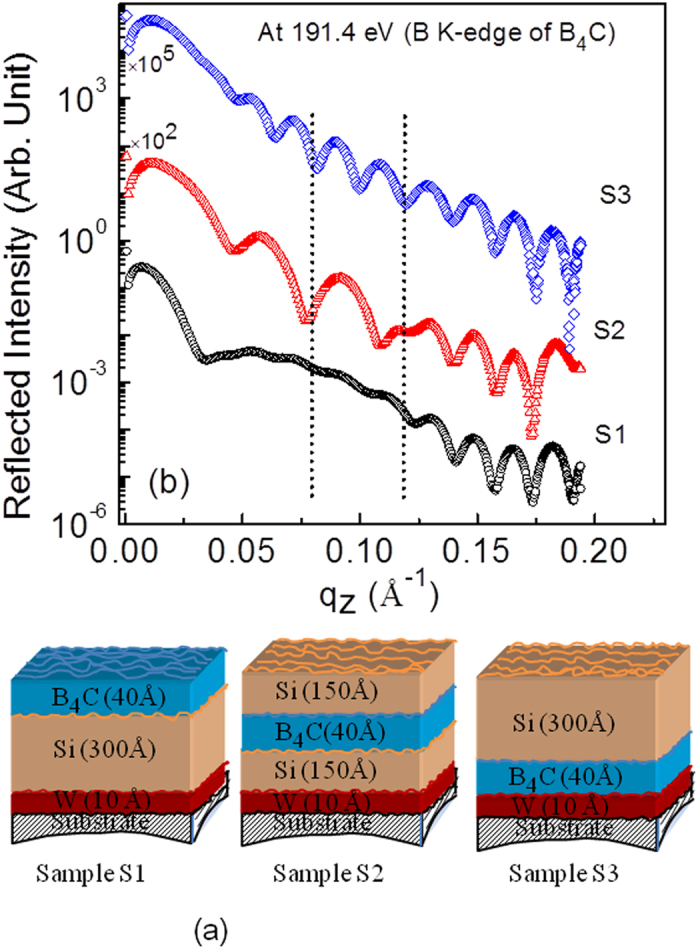
(**a**) Schematic of three fabricated samples with varying spatial positions of B_4_C layer. Surface roughness is represented by the undulating lines. (**b**) Measured R-SoXR profiles at a selected energy of 191.4 eV (B K-edge of B_4_C).

**Figure 3:**
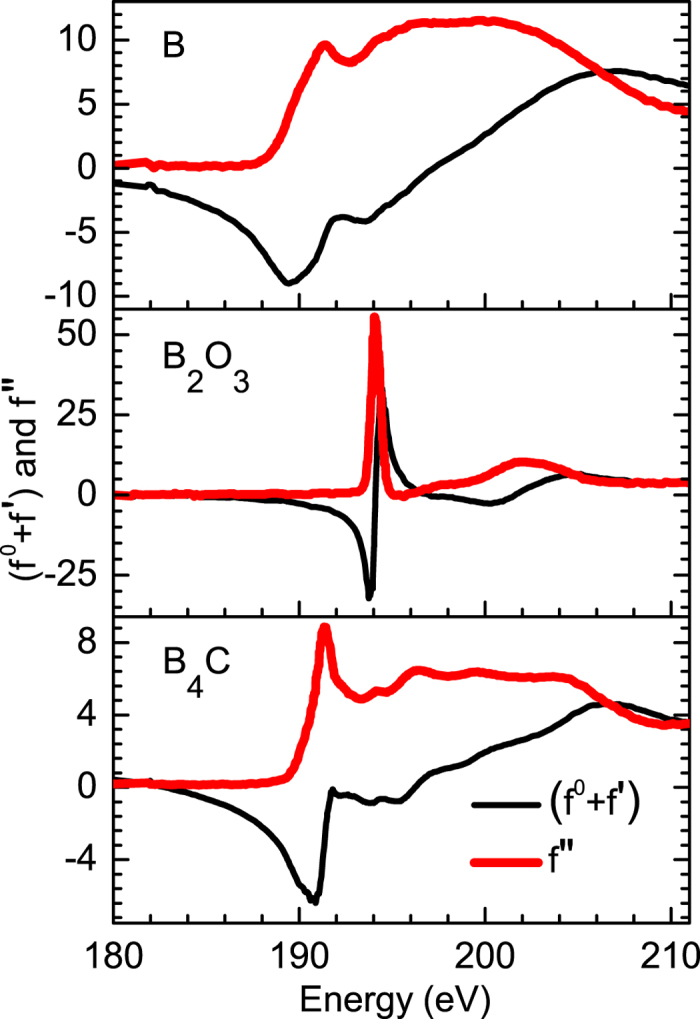
Measured ASF of B, B_4_C and B_2_O_3_ near boron K-edge to understand and correlate with the observed R-SoXR profiles.

**Figure 4:**
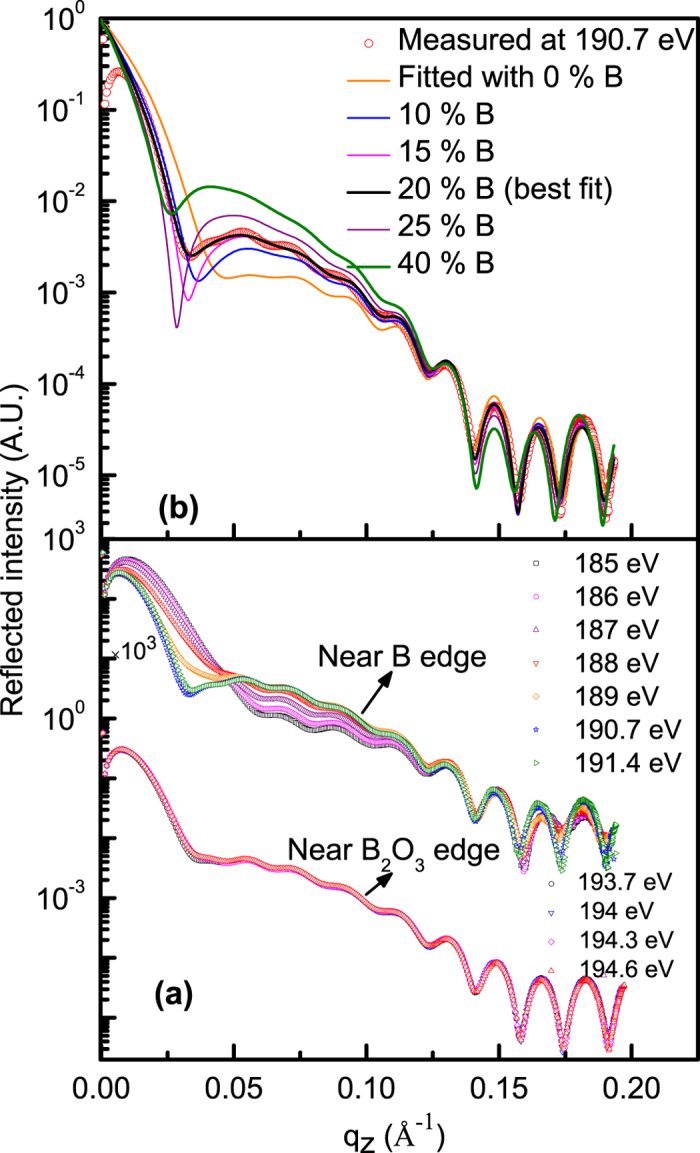
(**a**) Measured R-SoXR profiles of sample S1 at selected energies near B K-edge of both B and B_2_O_3_. (**b**) Measured R-SoXR profile of S1 at a selected energy of 190.7 eV (near B edge) along with fitted profiles with varying atomic percentage of B in the B_4_C layer.

**Figure 5:**
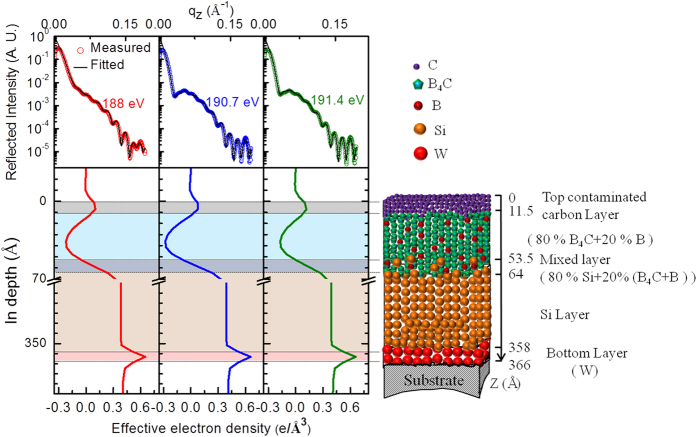
Top panel shows measured R-SoXR profiles along with best-fit data of Sample S1 at selected energies near B K-edge of elementary boron. The corresponding bottom panel shows effective EDP. The schematic at the right side shows the vertical depth profile of the composition modeled for the real structure in sample S1. Size of balls does not scale to the actual size of atoms and compounds.

**Figure 6:**
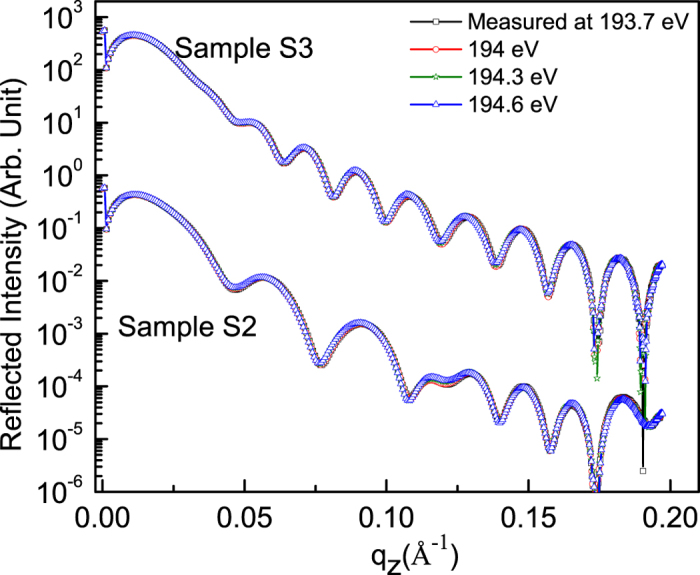
Measured R-SoXR profiles at selected photon energies near the B K-edge of B_2_O_3_ of samples S2 and S3.

**Figure 7:**
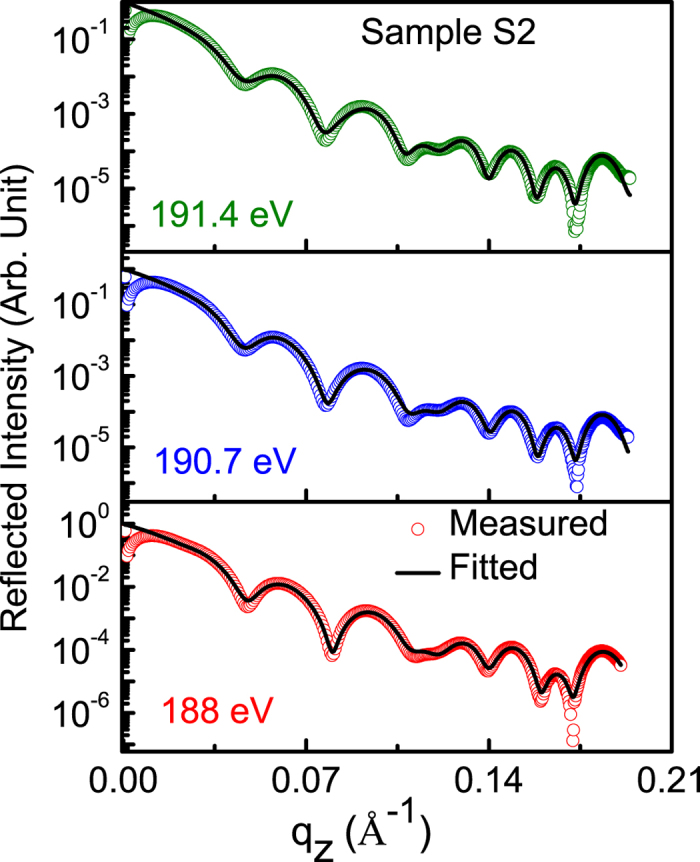
Measured R-SoXR curves along with best-fit profiles of sample S2 at selected energies near the B K-edge of elementary B.
