# Erratum: Determining Chemically and Spatially Resolved Atomic Profile of Low Contrast Interface Structure with High Resolution

**DOI:** 10.1038/srep31016

**Published:** 2016-08-25

**Authors:** Maheswar Nayak, P. C. Pradhan, G. S. Lodha, A. Sokolov, F. Schäfers

Scientific Reports 5: Article number: 8618; 10.1038/srep08618published online: 03 02 2015; updated: 08 25 2016

In this Article, A. Sokolov & F. Schäfers are incorrectly listed as being affiliated with ‘Indus Synchrotrons Utilization Division, Raja Ramanna Centre for Advanced Technology, Indore-452013 (M P), India’. The correct affiliation is listed below:

Helmholtz-Zentrum Berlin (HZB) BESSY II, Institute f. Nanometre Optics and Technology, Albert-Einstein-Strasse 15, D-12489 Berlin, Germany.

